# Genome-Wide Identification of the *AP2/ERF* Gene Family and Functional Analysis of *GmAP2/ERF144* for Drought Tolerance in Soybean

**DOI:** 10.3389/fpls.2022.848766

**Published:** 2022-03-28

**Authors:** Haitang Wang, Danqing Ni, Jiacheng Shen, Sushuang Deng, Huidong Xuan, Congcong Wang, Jianyu Xu, Li Zhou, Na Guo, Jinming Zhao, Han Xing

**Affiliations:** National Center for Soybean Improvement, Key Laboratory of Biology and Genetics and Breeding for Soybean, State Key Laboratory for Crop Genetics and Germplasm Enhancement, Ministry of Agriculture, College of Agriculture, Nanjing Agricultural University, Nanjing, China

**Keywords:** transcription factors (TFs), drought, soybean, overexpression, expression analysis, *AP2/ERF* gene family

## Abstract

Drought is a major environmental constraint that causes substantial reductions in plant growth and yield. Expression of stress-related genes is largely regulated by transcription factors (TFs), including in soybean [*Glycine max* (L.) Merr.]. In this study, 301 *GmAP2/ERF* genes that encode TFs were identified in the soybean genome. The TFs were divided into five categories according to their homology. Results of previous studies were then used to select the target gene *GmAP2/ERF144* from among those up-regulated by drought and salt stress in the transcriptome. According to respective tissue expression analysis and subcellular determination, the gene was highly expressed in leaves and encoded a nuclear-localized protein. To validate the function of *GmAP2/ERF144*, the gene was overexpressed in soybean using *Agrobacterium*-mediated transformation. Compared with wild-type soybean, drought resistance of overexpression lines increased significantly. Under drought treatment, leaf relative water content was significantly higher in overexpressed lines than in the wild-type genotype, whereas malondialdehyde content and electrical conductivity were significantly lower than those in the wild type. Thus, drought resistance of transgenic soybean increased with overexpression of *GmAP2/ERF144*. To understand overall function of the gene, network analysis was used to predict the genes that interacted with *GmAP2/ERF144*. Reverse-transcription quantitative PCR showed that expression of those interacting genes in two transgenic lines was 3 to 30 times higher than that in the wild type. Therefore, *GmAP2/ERF144* likely interacted with those genes; however, that conclusion needs to be verified in further specific experiments.

## Introduction

Plants are regularly exposed to environmental stresses during their life cycle that include drought, low temperature, saline alkali, diseases, and insect pests (Bhat et al., 2020). Under pressure from environmental stresses, plants evolved and formed complex signal transduction networks (Li et al., 2020). In a signal transduction network, various stress signals are identified and then expression of downstream related-genes is stimulated, allowing plants to survive in complex environments. Under stress conditions, transcription factors (TFs) have key roles in expression and regulation of functional genes. Transcription factors, also known as trans-acting factors, are DNA proteins that specifically bind to *cis*-acting elements in the promoter region of eukaryotic genes and activate or inhibit expression of downstream genes. In the Arabidopsis genome, more than 1,500 genes encode TFs, including those involved in regulating plant stress responses, such as AP2/ERF, bZIP, NAC, WRKY, and MYB (Mizoi et al., 2012).

Jofuku et al. (1994) isolated the first *AP2* gene from the model plant *Arabidopsis thaliana*, which is associated with flower development and contains two AP2/ERF (Apetala2/ethylene responsive factor) domains. Ohme-Takagi and Shinshi (1995) isolated ethylene response element binding proteins (ERF1, 2, 3, 4) from *Nicotiana tabacum* L., which contain a conserved ERF domain. Kagaya et al. (1999) isolated full-length cDNA sequences of RAV1 and RAV2 genes from *Arabidopsis thaliana*, which encode proteins that contain an *AP2/ERF* domain and a *B3* domain. Subsequently, in-depth research has been conducted on the *AP2/ERF* gene family in many species (Hu et al., 2020). Faraji et al. (2020) used RNA-seq analysis to conduct a detailed study on *tpap2s/ERFs* in the genome of durum wheat (*Triticum turgidum* ssp. *durum*) and identified 271 genes. They further analyzed the potential of the genes to affect resistance to drought and salt stress (Faraji et al., 2020). Li et al. (2020) identified 178 *AP2/ERF* genes in eggplant. Eggplant RNA-seq data on anthocyanin biosynthesis were combined with yeast single-hybridization and double-luciferase analysis to determine that *smap2/ERF* genes (*Smechr0902114.1* and *Smechr1102075.1*) were involved in regulation of anthocyanin biosynthesis (Li et al., 2021). Zhou and Yarra (2021) used genome-wide analysis to identify 172 *EgAP2/ERF*s (AP2, ERF, RAV, and Soloist subfamily members) in oil palm. Quantitative PCR analysis validated abiotic stress (salinity, cold, and drought)-responsive *AP2/ERFs* in the oil palm genome (Zhou and Yarra, 2021).

The AP2/ERF TFs are composed of four main functional domains: DNA binding domain, transcriptional regulatory domain, oligomerization site, and nuclear localization signal region. Members of this family contain one or more *AP2/ERF* domains (Jofuku et al., 1994). The domain is composed of 60 highly conserved amino acid sequences, which can recognize and bind DNA. Each *AP2/ERF* binding domain contains two conserved amino acid sequences, YRG and RAYD elements. An extremely basic element, YRG is composed of 19 to 22 amino acid residues, and it contains conserved YRG amino acid motifs. In addition, this region contains three β-foldings that are important in identifying various *cis*-acting elements, which is in the second β-folding. The difference between the 14th and 19th amino acid residues in the folding determines the specific binding of these TFs to different *cis*-acting elements. The RAYD element contains 42 to 43 amino acid residues, and the core sequence is composed of 18 highly conserved amino acid residues, which can form an amphiphilic α-helix that is involved in the interaction between *AP2/ERF* TFs and those of other genes (Yamasaki et al., 2008).

Soybean (*Glycine max* (L.) Merr.) provides edible protein and vegetable oil for human consumption (Holle and Damme, 2015) and is a crop with high economic value. As research on soybean genetics and genomics has increased, research on the *AP2/ERF* gene family has also gradually increased. For example, *GmERF057* can improve soybean resistance to salt and pathogens (Zhang et al., 2008), and *GmERF3* regulates soybean biological and abiotic stress responses (Zhang et al., 2009). The gene *GmDREB2* participates in abscisic acid (ABA)-dependent and independent signaling pathways and can induce expression of downstream genes such as *RD29A* and *COR15a* to increase soybean resistance to drought and high-salt environments (Chen et al., 2007). However, few studies have conducted genome-wide analysis of the *AP2/ERF* gene family. Simultaneously with the continuous improvement in information on the soybean genome, predicted gene numbers and gene structures have also changed. Therefore, it is necessary to analyze and identify members of the soybean *AP2/ERF* gene family (Mizoi et al., 2012).

In this paper, members of the soybean *AP2/ERF* gene family were identified and classified by using an HMM profile (pf00847) database (Jin et al., 2014). Then, the genes were analyzed and annotated. Based on published transcriptome data on salt tolerance and drought resistance, target genes were screened by transcriptome analysis, and then, stable transformation of soybean was performed. The gene improved drought resistance of transgenic soybean under stress. Thus, this study provides an important theoretical basis to understand the functions of *AP2/ERF* genes in soybean. Because of the increasing negative effects of stress on crop growth and development, a stress-resistance gene from the soybean genome was cloned in this study in order to provide an important gene resource and theoretical basis to improve stress resistance through genetic engineering (Xie et al., 2019). However, mechanisms by which plant cells sense and transmit stress signals and regulate downstream genes under drought stress are not clear. Therefore, it is very important to study *AP2/ERF* encoded TFs under drought stress in order to better understand drought resistance response mechanisms and ultimately cultivate drought-resistant soybean varieties. Such investigations will help further studies on roles of members of this gene family in soybean growth and development, stress response, and production breeding (Mizoi et al., 2012).

## Materials and Methods

### Identification of Soybean *GmAP2/ERF* Superfamily Members

To identify all *AP2/ERF* genes in soybean, the AP2/ERF HMM profile (PF00847) was used as a query (Chen et al., 2020; Faraji et al., 2020). After removal of redundant and incomplete ORF sequences, the SMART database^1^ was used to eliminate sequences that did not contain a complete *AP2/ERF* domain. Molecular weight (MW), isoelectric point (pI), and amino acid (aa) number of *GmAP2/ERF* proteins were obtained from ExPASy^2^ online tools. The software CELLO^3^ was used to predict *GmAP2/ERF* gene subcellular localization.

### Gene Structure, Conversed Motif, and Phylogenetic Analysis

The software TBtools (v1.0692) was used to draw gene structures by comparing cDNA sequences with the corresponding genomic DNA sequences of their *GmAP2/ERF* transporter members (Wang et al., 2017; Huang et al., 2021).

The online motif-finding tool MEME 4.11.2^4^ was used to identify conserved motifs in TFs of *AP2/ERF* genes. Parameters were as follow: 6–200, optimum width of amino acids; 25, maximum number of motifs; and 0 or 1 single motif in each sequence of the model.

All sequences of proteins encoded by *GmAP2/ERF* genes and those of 29 previously reported GmAP2/ERF transporters from *Arabidopsis thaliana* were used for multiple sequence alignments in MAFFT 7.0^5^. An unrooted phylogenetic tree was constructed using the neighbor-joining method in MEGA 6 with the following parameters: bootstrap value of 1,000, Poisson correction, and pairwise deletion (He et al., 2016; Li et al., 2021).

### Chromosomal Location and Duplication of *GmAP2/ERF* Genes

According to the soybean genome annotation file, the 300-kb hereditary interval gene densities were obtained and then further transformed into a gradient-colored heat map on soybean chromosomes or scaffolds. Tbtools was used to display chromosomal locations of *GmAP2/ERF* genes (Tian et al., 2020). Gene duplication events were analyzed by MCScanX in Tbtools and visualized in CIRCOS using default parameters. To evaluate selection pressure, ratios of non-synonymous (Ka) substitutions to synonymous (Ks) substitutions of each duplicated *GmAP2/ERF* gene were calculated by the NG method in TBtools. Values of Ks > 2.0 were discarded to avoid saturation of substitutions. Occurrence time of duplicated *GmAP2/ERF* gene pairs was calculated as follows: *T* = Ks/(2λ × 10^–6^), where λ = 6.5 × 10^–9^ (Blanc and Wolfe, 2004; Zhou and Yarra, 2021).

### Plant Material, Growth Conditions, and Drought Stress Treatments

Soybean cultivars Qihuang22 and Tianlong No.1 were used in different experiments. Qihuang22 was used to isolate the *GmAP2/ERF144* gene, and Tianlong 1 (wild type, WT) was the recipient parent to develop overexpression (OE) lines. Plants were grown in a controlled-environment growth chamber at 26/23°C and 70% relative humidity under a 16-h light/8-h dark photoperiod. Fourteen-day-old soybean seedlings were subjected to drought stress by withholding water supply. Root, stem, and leaf samples were harvested at 0, 2, 4, 6, and 8 d following drought treatment of seedlings, with samples subsequently frozen quickly in liquid nitrogen (Borges et al., 2012; Yang C. et al., 2020; Wang et al., 2021).

To determine whether *GmAP2/ERF144* could regulate the drought response of soybean, three groups of experiments were conducted. Two strains with good drought performance were selected for the test. Steps in the process of transgenic verification of *GmAP2/ERF144* are presented in Supplementary Figure 4. Wild-type Tianlong 1 and *GmAP2/ERF144* overexpression lines were planted under the same conditions, and drought tests were conducted when soybean compound leaves with three leaflets were fully unfolded (approximately 2 weeks). Three types of drought tests were conducted: (1) a single plant of each soybean type (WT, OE1, OE2) was dry for 18 d and then rehydrated for 7 d; (2) after 16 d of drought, two plants of each soybean type (WT, OE1, OE2) were rehydrated for 7 d; and (3) a single plant of each soybean type (WT, OE1, OE2) naturally dry for 28 d was rehydrated for 7 d.

### Reverse-Transcription Quantitative Polymerase Chain Reaction

Total RNA was isolated from soybean roots, stems, and leaves using the protocol of an RNAprep Pure Plant Kit (Tiangen, Beijing, China). Purity and concentration of total RNA were determined using a Nanodrop UV spectrophotometer and an RNA Nano chip on an Agilent Bioanalyzer 2100, respectively. The cDNA was synthesized using a Prime Script™ RT Reagent Kit (TaKaRa, Dalian, Japan) following a standard protocol. Reverse-transcription quantitative PCR (RT-qPCR) was performed for each cDNA template using AceQ qPCR SYBR Green Master Mix (Vazyme, Nanjing, China) according to a standard protocol. Amplification conditions for PCRs were 95°C for 3 min followed by 40 cycles of 95°C for 10 s and 60°C for 30 s. In RT-qPCR assays, three biological replications were used, and three measurements were performed on each replicate. Results from PCRs were normalized using the Ct value corresponding to the soybean actin gene *GmActin11* (*Glyma.18g290800*) as the internal control. Specificity of reactions was verified by melting curve analysis, and relative mRNA level for each gene was calculated as follows: ratio = 2^–ΔΔ*Ct*^ = 2^–[*Ct,t–Ct,r*]^ (*Ctcycle* threshold: *Ct*, *tCt* of the target gene; *Ct*, *rCt* of the control gene) (Huang et al., 2019). The NCBI Primer BLAST was used to design all primers (Supplementary Table 2). Reverse-transcription qPCR was conducted on a Light Cycler 480 instrument (Bessire et al., 2011).

### Subcellular Localization of Protein Encoded by *GmAP2/ERF144*

To determine subcellular location of the protein encoded by *GmAP2/ERF144*, first, cNLS online software^6^ was used to analyze the amino acid sequence and then predict the nuclear localization signal (NLS) of *GmAP2/ERF144*. To further verify localization of the protein encoded by *GmAP2/ERF144* within plant cells, the conserved domain sequence of the *GmAP2/ERF144* gene without the terminator was cloned into a pBIN-GFP4 vector to construct a fusion vector, following the manufacturer’s protocol (Supplementary Figure 2). When sequencing was successful, constructs and empty bodies were transiently transformed into tobacco leaves. Leaf fluorescence was observed under a Zeiss LSM 880 Upright Confocal Microscope (Carl Zeiss, Thornwood, NY, United States) at 48 to 60 h post-inoculation. Excitation wavelengths were 488 nm for green fluorescent protein (GFP) and 405 nm for 4′,6-diamidino-2-phenylindole (DAPI; Yang et al. 2020).

### Construction of *GmAP2/ERF144* Plant Overexpression Vectors and Plant Transformation

The full-length coding region of *GmAP2/ERF144* was cloned into a *pTF101.1* vector for overexpression of *GmAP2/ERF144* under the control of *CaMV 35S* promoter (35S:GmAP2/ERF144). Primers are listed in Supplementary Table 2. The 35S:GmAP2/ERF144 construct and *pTF101.1* empty vector control were individually transformed into *A. tumefaciens* strain *EHA101 via* electroporation. Tian Long No. 1 was used for tissue culture and transformation according to a previously reported protocol (Huang et al., 2019). The T3 homozygous lines were used for phenotypic investigation. To confirm the transgenic lines, PCR and RT-qPCR were used to verify *GmAP2/ERF144* overexpression in transgenic plants. The basta was also used to screen soybean seedlings with three fully expanded compound leaves.

### Network Prediction of Genes Interacting With the *GmAP2/ERF144* Gene

To predict the proteins interacting with *GmAP2/ERF144*, each protein was separately submitted to the STRING database^7^. Online prediction software displayed both experimentally demonstrated and hypothetical proteins interacting with *GmAP2/ERF144* (Noman et al., 2019). Primers are listed in Supplementary Table 2.

## Results

### Genome-Wide Identification, Phylogenetic Analysis, and Classification of *GmAP2/ERF* Members

**FIGURE 1:**
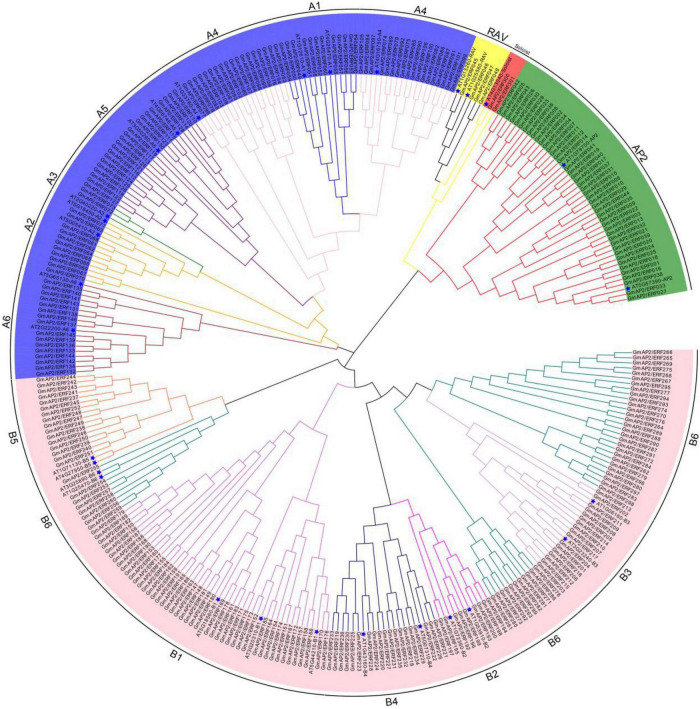
Phylogenetic tree of GmAP2/ERF proteins in soybean and Arabidopsis. Five subfamilies are highlighted by different colors. Arabidopsis is marked with a blue pentagram.

To identify all *AP2/ERF* genes in soybean, the AP2/ERF HMM profile (PF00847) was used as a query. In the HMM search of the *G. max* genome, 301 *GmAP2/ERF* genes were identified. The *GmAP2/ERF* genes were named in sequence from *GmAP2/ERF001* to *GmAP2/ERF301* by following the classification principles/criteria of *A. thaliana* (Supplementary Table 1). Length of amino acid sequences of GmAP2/ERF proteins varied from 144 aa to 710 aa, with corresponding molecular weights ranging from 15,961.1 Da to 77,512.2 Da. Predicted theoretical points (pI) ranged from 4.26 to 11.66. To classify GmAP2/ERF proteins, full-length sequences of the 301 GmAP2/ERF proteins and those of 29 AtAP2/ERF proteins of Arabidopsis were aligned, and a phylogenetic tree was constructed using the neighbor-joining method (Figure 1).

According to the phylogenetic tree and homology, the 44 proteins containing two AP2 domains were closely related to the AP2 subfamily of proteins. Four proteins containing one AP2 domain and one B3 domain were classified in the RAV subfamily of proteins. However, 98 proteins were classified in the DREB subfamily of proteins and 153 proteins were classified in the ERF subfamily of proteins. *GmAP2/ERF300* and *GmAP2/ERF301* showed homology with *AT4G13040* and therefore were classified as members of the Soloist subfamily of proteins. Additionally, according to the classification criteria of *A. thaliana* ERF and DREB subfamilies, the corresponding subfamilies in *G. max* could be divided into six groups (Figure 1). The DREB subfamily had 12, 11, 2, 36, 21, and 16 members in the A1, A2, A3, A4, A5, and A6 subgroups, respectively, and the ERF subfamily had 45, 9, 17, 19, 16, and 47 members in the B1, B2, B3, B4, B5, and B6 subgroups, respectively. Thus, DREB and ERF subfamilies of *GmAP2/ERF* encoded proteins were dominant in *G. max*.

### Structure and Motif Analysis of *GmAP2/ERF* Genes

To examine the structural diversity of *GmAP2/ERF* genes, number of exon–introns and distribution of conserved domains of *GmAP2/ERF* genes were investigated. Number of introns among different *GmAP2/ERF* subfamilies varied markedly. Most *DREB* and *ERF* genes did not have introns. All *AP2* and *Soloist* genes had from three to nine introns, whereas in the *RAV* subfamily, genes did not contain introns. The *GmAP2/ERF* genes classified in the same subfamily showed similar gene structures.

To better illustrate conserved domain patterns of *GmAP2/ERF* gene members, MEME was used. The *GmAP2/ERF* genes within the same subfamily displayed similar motif compositions. Most *AP2* subfamily members contained motif-10, motif-2, motif-3, and motif-5, whereas *ERF* subfamily members contained motif-6 and motif-5. Most *DREB* subfamily members contained motif-2 and motif-5, whereas *RAV* subfamily genes contained motif-7, moti-8, motif-1, and moif-4 (Supplementary Figures 1A–D).

### Chromosomal Location Analysis and Duplication of *GmAP2/ERF* Genes

**FIGURE 2:**
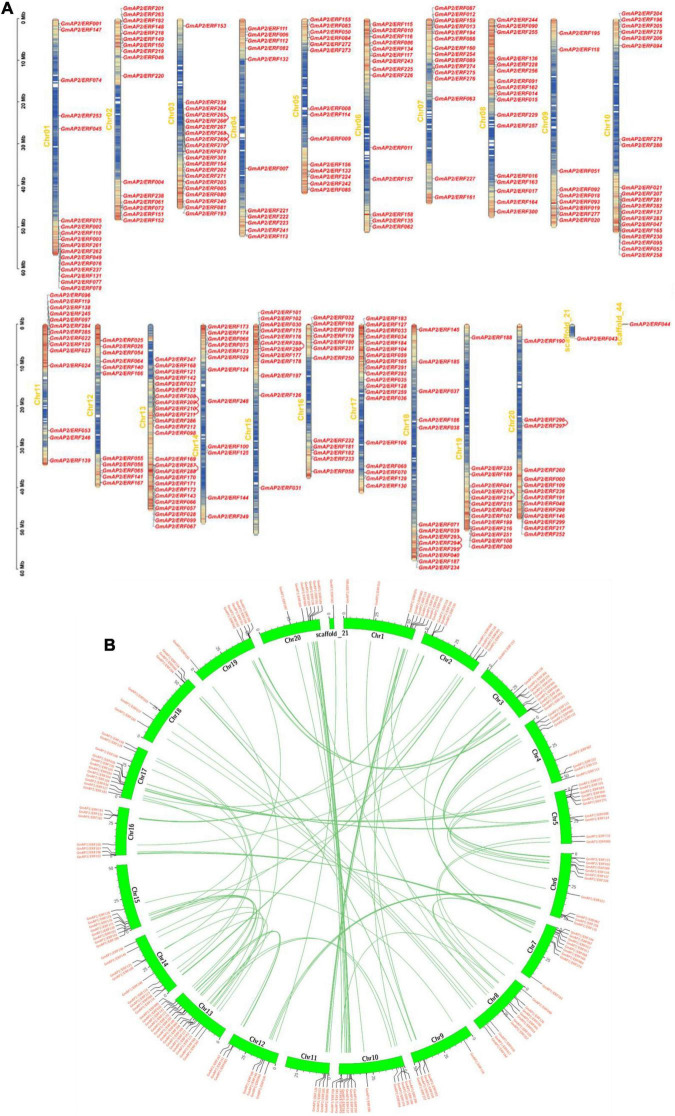
Chromosomal distributions of *GmAP2/ERF* genes and schematic of interchromosomal relations. **(A)** Chromosomal distributions of *GmAP2/ERF* genes. Chromosomal (Chr) names in yellow are on the left, and gene names are on the right. Left-side scale is in megabases (Mb). Gradient colors from red (high) to blue (low) indicate gene density in heat maps on soybean chromosomes, by setting the estimated hereditary interval as 300 kb. Red indicates high gene density, and blue indicates low gene density. **(B)** Schematic of interchromosomal relations of soybean *GmAP2/ERF* genes. Green lines indicate all syntenic blocks in the soybean genome. Heat maps represent the gene density.

The 301 *GmAP2/ERF* genes were mapped on the 20 chromosomes and scaffold_21 and scaffold_44 of the soybean genome. The highest number of genes was 25 on Chr13, whereas scaffold_21 and scaffold_44 each contained one gene. By contrast, Chr09 contained the fewest genes (only nine). There were 11 genes on Chr04 and Chr12 and 14 genes on Chr05, Chr06, Chr11, and Chr19. Coincidentally, there were 12 genes on adjacent chromosomes 14, 15, and 16. There were 17 genes distributed in the scaffolds of Chr01 and Chr08. The distribution of the 301 genes on the 20 chromosomes was relatively balanced (Figure 2A).

Gene duplication is an important event leading to amplification of gene families. To elucidate the mechanism of expansion of *GmAP2/ERF* genes, gene duplication event analysis was performed, and details of duplicated gene pairs are presented in Figure 2B. A chromosomal region within 200 kb containing two or more genes is defined as a tandem duplication event. In this study, six tandem duplication events associated with four *GmAP2/ERF* genes (*GmAP2/ERF209*/*GmAP2/ERF208*, *GmAP2/ERF210/GmAP2/ERF208*, *GmAP2/ERF208/GmAP2/ER F211*, *GmAP2/ERF210/GmAP2/ERF209*, *GmAP2/ERF211*/*GmAP2/ERF209*, *GmAP2/ERF211*/*GmAP2/ERF210*) were detected on Chr14. By contrast, segmental duplications result in a large amount of duplicated chromosomal blocks in a genome and often occur during polyploidization events with chromosome rearrangements. In the soybean genome, 96 segmental duplication events associated with 180 *GmAP2/ERF* genes were identified (Figure 2B). Genes that undergo tandem and segmental duplication events are closely related genetically, and thus, these results provide a potential reference for functional prediction.

### *Cis*-Element Analyses of Soybean *GmAP2/ERF* Genes

The *cis*-elements in promoter regions are important in regulating gene transcription and abiotic stress responses. Therefore, promoter region sequences (i.e., 2-kp upstream sequences from gene initiation codons) of *GmAP2/ERF* genes were subjected to *cis*-element analysis. *Cis*-elements included ABA-responsive elements (ABRE), drought-inducible elements (MBS, MYB binding site), low-temperature responsive elements (LTR), MEJA-responsive elements (CGTCA-motif), SA-responsive elements (TCA-element), and defensive and stress responsive elements (TC-rich repeats). *Cis*-elements are displayed by proportion in Supplementary Figure 2. Among the elements, ABRE, MBS, and CGTCA-motif were detected in almost every promoter region of the *GmAP2/ERF* genes. However, correlations between *cis*-elements and gene responses to abiotic stresses need further experimental validation to determine whether *GmAP2/ERF* genes respond abiotic stresses.

### Expression Profiling of the *GmAP2/ERF* Genes in Soybean During Saline Stress and Drought

Genes in the *AP2/ERF* family can be induced by various abiotic stresses in some species. To determine expression of *GmAP2/ERF* genes in response to abiotic stress, *GmAP2/ERF* gene expression profiles were examined in soybean under saline stress and drought by using previously published RNA-seq data^8^. Based on RNA-seq results, 152 and 112 *GmAP2/ERF* genes responded to drought and saline stress, respectively. Of those genes, 67.1% (102/152) and 81.25% (91/112) were up-regulated under drought and saline stress, respectively. Therefore, 50 and 21 genes were down-regulated under drought and saline stress, respectively.

**FIGURE 3:**
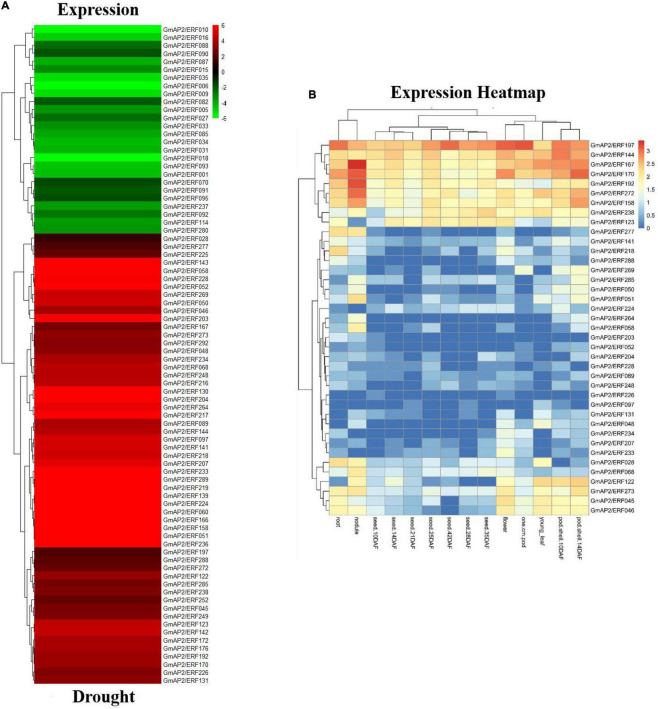
Expression of drought-responsive differentially expressed genes and expression profiles of *GmAP2/ERF* genes in different soybean tissues. **(A)** Heat map with hierarchical cluster analysis of drought-responsive differentially expressed soybean *GmAP2/ERF* genes. Red indicates high relative transcript abundance, whereas green indicates low relative transcript abundance. **(B)** Heat map of expression profiles of soybean *GmAP2/ERF* genes in 14 soybean tissues. Hierarchical clustering of the 39 soybean *GmAP2/ERF* genes was constructed by average linkage with Euclidean distance in MeV 4.9 software. Different colors indicate relative transcript abundance of soybean *GmAP2/ERF* genes in 14 soybean tissues, with red indicating high abundance and blue indicating low abundance.

Thirty-nine genes were up-regulated under both abiotic stresses, of which six were significantly up-regulated, including *GmAP2/ERF192*, *GmAP2/ERF272*, *GmAP2/ERF158*, *GmAP2/ERF167*, *GmAP2/ERF144*, and *GmAP2/ERF197*. Tissue expression data of the 39 genes was downloaded from the SoyBase database (see text footnote 8). The six genes had relatively high expression in 14 tissues of soybean but particularly in roots, stems, and leaves (Figure 3 and Supplementary Figure 3).

**FIGURE 4:**
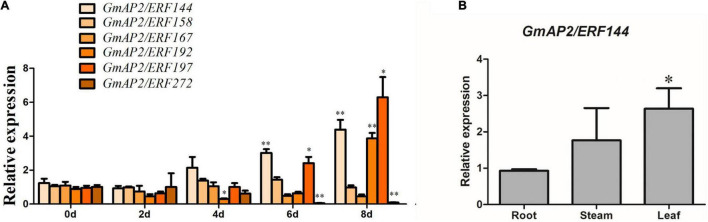
Reverse-transcription quantitative PCR analyses of expression of six representative soybean *GmAP2/ERF144* genes during increasing time of drought and gene expression pattern in different soybean tissues. **(A)** Reverse-transcription qPCR analyses of the expression of six representative genes in the *GmAP2/ERF* family during drought stress treatment. Leaves of two-week-old soybean plants were collected for analysis when watering was stopped for 0, 2, 4, 6, and 8 d. **(B)** Tissue expression pattern of gene *GmAP2/ERF144*. Values are the mean ± SE. *p < 0.05; ^**^p < 0.01 (Student’s *t*-test).

According to previous studies, the six stress-related genes selected from the *ERF* family analysis were subjected to drought treatment in soybean seedlings. The RNA was extracted from soybean leaves at 0, 2, 4, 6, and 8 d of drought treatment. Expression patterns of the candidate genes under drought stress were investigated by fluorescence quantitative PCR, with actin used as the internal reference gene. Genes viz., *GmAP2/ERF197*, *GmAP2/ERF192*, and *GmAP2/ERF144* was up-regulated four to seven times after 8 d of drought treatment (Figure 4A). With an increase in exposure to drought, the expression of *GmAP2/ERF144* also increased, ultimately increasing by more than four times (Figure 4A). Because expression of *GmAP2/ERF144* was more responsive to drought than that of other genes, it was selected for stable transformation of soybean.

To analyze expression of *GmAP2/ERF144* in different organs, fluorescence quantitative PCR technology was used. The RNA extracted from roots, stems, and leaves of soybean seedlings was used as a template, and actin was the internal reference gene. Expression of *GmAP2/ERF144* mRNA was the lowest in roots, with expression approximately 1.5 times higher in stems and approximately 2.5 times higher in leaves (Figure 4B).

### Subcellular Localization of *GmAP2/ERF144*

**FIGURE 5:**
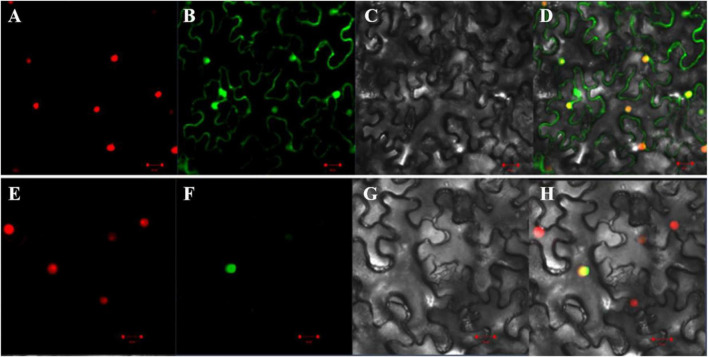
Subcellular localization of GmAP2/ERF144. **(A)** 35S::PIP2;1-mCherry fusion protein. **(B)** 35S::EGFP fusion protein. **(C)** Bright-field. **(D)** Merged image of panels **(A–C)**. **(E)** 35S::PIP2;1-mCherry fusion protein. **(F)** 35S::GmAP2/ERF144-EGFP fusion protein. **(G)** Bright-field. **(H)** Merged image of panels **(E–G)**. Bar = 0.02 mm.

To determine subcellular localization of the GmAP2/ERF144 protein, first, the amino acid sequence was analyzed, and protein domain and nuclear localization signal (NLS) were predicted using online software^9^. The coding region of the *GmAP2/ERF144* gene was 939 bp in length, and it encoded a protein containing 312 amino acids. Amino acid sequence analysis showed that the GmAP2/ERF144 protein contained an AP2 domain located at 150–213 amino acid residues (Supplementary Figure 4A) and an NLS (lcriykkha) at 248–258 amino acid residues (Supplementary Figure 4B). In the subcellular localization analysis, the *GmAP2/ERF144* full-length CD without stop codon was cloned, and expression vectors 35S:*GmAP2/ERF*144:GFP (containing *GmAP2/ERF144*) and 35S:GFP (without *GmAP2/ERF144*) were constructed. The two carriers were transformed into tobacco leaves, and a laser scanning confocal microscope was used to detect fluorescence signals. The 35S:GFP green fluorescence was observed in cell membrane, nucleus, and endoplasmic reticulum, whereas the 35S:GmAP2/ERF144 fusion protein was only concentrated in the nucleus (Figure 5). Therefore, the GmAP2/ERF144 protein was a nuclear localized protein.

### Overexpression of *GmAP2/ERF144* Gene Improved Drought Tolerance of Transgenic Plants

**FIGURE 6:**
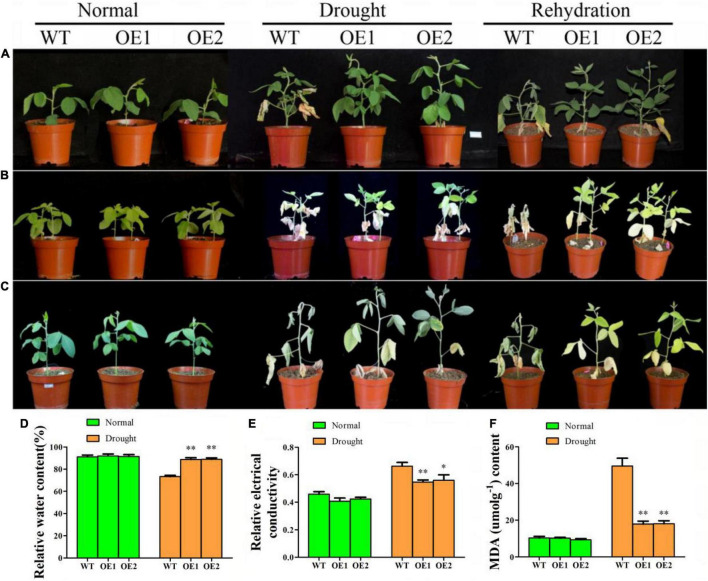
The *GmAP2/ERF144* acts as a positive regulator in response to drought stress. **(A)** Performance of wild-type (WT), and two *GmAP2/ERF144* overexpressing lines (OE1 and OE2) under drought stress. Two-week-old plants were incubated at 18 days drought stress, and rehydration after drought. **(B)** Performance of wild-type (WT), and two *GmAP2/ERF144* overexpressing lines under drought stress. Two-week-old plants were incubated at 16 days drought stress, and rehydration after drought. **(C)** Performance of wild-type (WT), and two *GmAP2/ERF144* overexpressing lines under drought stress. Two-week-old plants were incubated at 28 days drought stress, and rehydration after drought. **(D)** The leaf relative water contents in different lines under drought stress. **(E)** Leaf relative conductivity in different lines under drought stress. **(F)** MDA content in different lines under drought stress (*n* = 3). Data represent the mean ± SE. *, *P* < 0.05 (Student’s *t*-test). ^**^, *P* < 0.01 (Student’s *t*-test).

To determine whether *GmAP2/ERF144* could regulate the drought response of soybean, three groups of experiments were conducted. In the first single-plant drought rehydration test, survival of overexpressed lines was 100%, whereas growth of the wild type was weak and only 13.3% (4/30 plants) survived after rehydration (Figure 6A). With two soybeans in a small flowerpot, plants did not grow as well as a single plant in a flowerpot. After 16 d of drought, leaves of overexpressed lines turned yellow, whereas those of the wild type withered. Degree of wilting of overexpressed lines was significantly lower than that of the wild type. After 1 week of rehydration, all overexpression lines survived, whereas almost all wild-type plants died (29/30) (Figure 6B). After 28 d of natural drought, wild type plants did not survive (0/30), whereas survival of transgenic lines was 53% (16/30) (Figure 6C). Thus, expression of *GmAP2/ERF144* improved drought tolerance of transgenic plants.

Under normal watering, there was no morphological difference between leaves of the wild type and those of transgenic soybean, and there was no significant difference in leaf relative water content (Figure 6D). However, after 2 weeks of drought, wild-type plants suffered from the serious water shortage, and leaf relative water content decreased significantly compared with that of the two transgenic lines (Figure 6D).

To evaluate physiological changes in transgenic plants, malondialdehyde (MDA) content and relative conductivity of leaves in wild-type and transgenic soybeans were measured before and after drought treatment (Figures 6E,F). After 14 days of drought stress, MDA content in the wild type increased significantly by approximately five times that under normal conditions. In addition, MDA content of the wild type was significantly higher than that of transgenic lines by 2.5 times. There was no significant difference in leaf relative conductivity between the wild type and transgenic lines before treatment. After 2 weeks of drought, leaf relative conductivity of both the wild type and transgenic lines increased, but that of the wild type was significantly higher than that of overexpression lines. Therefore, drought resistance of soybean increased with overexpression of *GmAP2/ERF144*.

### Expression Analysis of Genes Interacting With *GmAP2/ERF144* and Regulatory Network in Soybean

**FIGURE 7:**
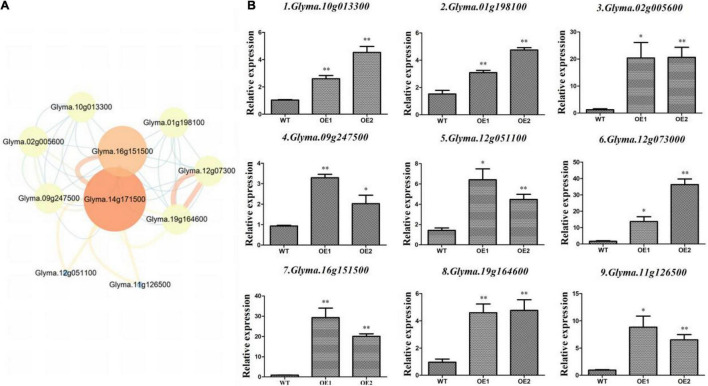
Expression of interacting genes in the regulatory network of *GmAP2/ERF144*. **(A)** STRING-predicted regulatory network of genes interacting with *GmAP2/ERF144* in soybean. **(B)** Expression profiles of nine genes (of 10) that interacted with *GmAP2/ERF144* in wild-type (WT) and overexpressing (OE) lines of soybean. Protein–protein interaction network of GmAP2/ERF144 was detected *in silico* using the STRING database (https://string-db.org/). Values are the mean ± SE, *n* = 3. *p < 0.05; ^**^p < 0.01 (Student’s *t*-test).

Overexpression lines of *GmAP2/ERF144* indicated the *GmAP2/ERF144* gene was associated with drought resistance in soybean. Therefore, to fully understand possible effects of the *GmAP2/ERF144* gene, the String database was used to predict its regulatory network. Ten genes were predicted to interact with *GmAP2/ERF144* (Figure 7A). Because there was no information on gene *Glyma14g34070*, it was not considered further. Total RNA from drought-treated WT and OE plants was isolated and reverse transcribed to cDNA, and gene-specific primers (Supplementary Table 2) were used to perform qPCR. Differential expression patterns of the nine genes in WT and OE plants are shown in Figure 7B.

Whereas *Glyma.10g013300* (*GmbZIP*) increases salt and freezing tolerance, most other ABA-induced *bZIP* genes increase drought tolerance. This divergence suggests functional specificity of each *bZIP* gene in improving plant stress tolerance. Alternatively, each group of *bZIP* genes may have specific functions. For example, group A *bZIP* genes are involved in ABA signaling and drought tolerance (Liao et al., 2008).

The gene *Glyma.01g198100* encodes a transcription factor bhlh18. Basic helix–loop–helix (bHLH) TFs are encoded by one of the largest gene families in plants, and they participate in various physiological processes. The *bHLHs* function in transcriptional regulation networks by binding to E-box elements within the promoter region of their target genes (Huang et al., 2013).

Compared with the wild type, *Glyma.16g151500* was up-regulated by 25 times in overexpression lines (Figure 7B), which was the greatest increase in expression among the predicted interaction genes. The gene encodes a protein containing a NAC domain and that has DNA binding characteristics. Overall, expression of the nine interacting genes was significantly up-regulated in the two lines overexpressing *GmAP2/ERF144* by 3 to 30 times compared with that in the wild type. Thus, the nine genes likely interacted with *GmAP2/ERF144*, although specific experiments are needed to verify that conclusion.

## Discussion

Plant growth and development under different environmental stresses are regulated by genetic elements. Genetic improvement of drought resistance has become an important focus of research in soybean breeding, particularly as drought increases. Transcription factors contain one or more specific DNA-binding domains and are essential in regulating gene expression throughout the life cycles of higher plants (Hao et al., 1998). The AP2/ERF (APETALA2/ethylene-responsive element binding factors) TFs are a large group that is mainly found in plants (Yang S. U. et al., 2020). The TFs of this family are important regulators of many biological and physiological processes, including plant morphogenesis, responses to various stresses, hormone signal transduction, and biosynthesis of metabolites (Feng et al., 2020).

Transcription factors in the AP2/ERF family are mainly classified into four major subfamilies, including DREB (dehydration-responsive element-binding), ERF (ethylene-responsive element-binding), AP2 (APETALA2), and RAV (related to ABI3/VP), with some also classified in the Soloist subfamily (a few unclassified factors) (Riechmann and Meyerowitz, 1998; Magnani et al., 2004). Transcription factors of the AP2/ERF superfamily regulate diverse processes involved in plant development and are also important in hormonal regulation and stress responses. Based on genome-wide sequence analyses of those TFs, 147 and 163 have been reported in *Arabidopsis thaliana* and rice, respectively (Nakano et al., 2006; Sharoni et al., 2011). Based on the EST database, 112, 167, 53, and 117 TFs in the AP2/ERF family have also been reported in tomato (Sharma et al., 2010), maize, barley, and common wheat (Jing et al., 2011), respectively. In this study, the AP2/ERF HMM profile (PF00847) was used as a query to identify the genes in the *AP2/ERF* family in soybean. The HMM search of the *G. max* genome resulted in the identification of 301 *GmAP2/ERF* genes. Based on the phylogenetic tree and homology analysis, 44 proteins that contained two AP2 domains were closely related to the AP2 subfamily of proteins, and four proteins that contained one AP2 domain and one B3 domain were classified in the RAV subfamily of proteins. However, 98 proteins were identified in the DREB subfamily of proteins, and 153 proteins were identified in the ERF subfamily of proteins (Supplementary Figure 1). The genes *GmAP2/ERF300* and *GmAP2/ERF301* showed homology with *AT4G13040*, and therefore, the proteins of those genes were classified as members of the Soloist subfamily of proteins.

Gene duplication might be the primary reason for expansion of the soybean *AP2/ERF* family (Figure 2B). Duplicate genes are an important feature of genome structure, and evolutionary processes such as duplication events, especially segmental and tandem repeats, have expanded members of gene families in plants (Abdullah et al., 2021; Musavizadeh et al., 2021). In addition, mutations in gene structure and at the promoter site cause diversity in functions of members of a gene family (Faraji et al., 2020). Overall, the *AP2/ERF* family genes in soybean may have been relatively highly influenced by evolutionary pressure (Musavizadeh et al., 2021).

In AP2/ERF TFs that contain the AP2 DNA-binding domain (approximately 60 amino acids in length), it directly interacts with *cis*-acting elements such as dehydration responsive elements (DRE)/C-repeat elements (CRT) and GCC-box at the promoter of target genes (Allen et al., 1998; Riechmann and Meyerowitz, 1998; Magnani et al., 2004; Mizoi et al., 2012; Yang S. U. et al., 2020). In this study, previously collected transcriptome data were used to screened six of the 39 genes up-regulated under drought and salt stress for drought-induction treatment (Figure 4 and Supplementary Figure 2). The genes *GmAP2/ERF197*, *GmAP2/ERF192*, and *GmAP2/ERF144* were up-regulated four- to seven-fold after 8 d of drought treatment (Figure 4). In this quantitative analysis, *GmAP2/ERF144* expression increased as the time of drought increased. Hence, although results are preliminary, *GmAP2/ERF144* likely responds to drought stress. Therefore, *GmAP2/ERF144* was overexpressed in soybean using an *Agrobacterium*-mediated method. In three types of drought tests, drought resistance of overexpression lines increased compared with that of wild-type soybean (Figure 6).

Water deficient conditions caused by drought limit plant growth and crop yields. The dehydration-responsive element/C-repeat (DRE/CRT) is recognized by the DREB subfamily of the AP2/ERF family. Members of the DREB subfamily function in regulating plant resistance to abiotic stress, including drought (Yamaguchi-Shinozakiaib and Shinozaki, 1994; Kole et al., 2015). According to Park et al. (2021) genes *DREB2A* and *DREB2B* are induced by both dehydration and high-salt stress, although they do not respond to low temperature in Arabidopsis (Park et al., 2021). Haake et al. (2002) isolated the *CBF4* gene, also named *DREB1D*, from Arabidopsis. The gene is important in drought adaptation and is induced by drought stress but not by low temperatures. Similarly, TaERF3 from tomato directly interacts with the GCC-box in the promoters of stress-related genes, including *BG3*, *Chit1*, *RAB18*, *LEA3*, *TIP2*, *POX2*, and *GST6*, to promote resistance to salt and drought stress in wheat (Min et al., 2014; Rong et al., 2014). The gene *GmDREB2* encodes a DRE-binding transcription factor in soybeans and is induced under drought and salinity stresses. Overexpression of *GmDREB2* in transgenic plants increases expression of downstream gene transcripts and content of free proline to increase tolerance to salinity and drought stresses (Chen et al., 2007). In this study, the GmAP2/ERF144 protein targeted the nucleus (Figure 5) and increased drought resistance of overexpressed plants following stable genetic transformation of soybean. The results in this study are consistent with those of Yamaguchi-Shinozakiaib and Shinozaki (1994).

Leaf water content is a major physiological indicator that reflects a plant’s ability to withstand adversity in stressful environments (Jin et al., 2017; Zhang et al., 2019). Heat and drought stress decrease plant water uptake and increase transpiration, which in turn significantly decrease efficiency of water utilization and moisture retention in leaves (Alhaithloul, 2019; Cohen et al., 2020). In the present study, physiological indices of the wild type and two overexpression lines were measured under natural culture conditions and drought treatment for 14 d. After 14 d of drought, relative water content of leaves of overexpression plants was significantly higher than that of leaves of the wild type (Figure 6D). Increased ability of OsERF115/AP2EREBP110-OE transgenic lines to utilize water efficiently may contribute to conserve plant water content and maintain a lower leaf temperature than that in WT plants under heat–drought combined stress (Park et al., 2021). Drought stress negatively affects many aspects of cellular physiology. The major responses to water deficit stress are ROS accumulation, membrane damage, and altered antioxidant enzymatic activity, which subsequently leads to the loss of membrane integrity. Electrical conductivity is negatively correlated with membrane integrity and reflects the extent of membrane injury. In the present study, under drought treatment, MDA content and EC were significantly lower than those in the wild type (Figures 6E,F). Therefore, the results suggested there was less production of ROS in response to water deficit in overexpression lines, which helped to maintain membrane integrity (Ying et al., 2015). The results of this study and others suggest that overexpression of *GmAP2/ERF144* contributes to improved drought stress tolerance by increasing water retention capability.

To fully understand the function of *GmAP2/ERF144*, network analysis was used to predict those genes that interacted with *GmAP2/ERF144* (Figure 7A). Interaction gene *Glyma.01g198100* encodes transcription factor bhlh18. Basic helix–loop–helix (bHLH) TFs are members of one of the largest gene families in plants, and they participate in various physiological processes. The bHLHs can function in a transcriptional regulation network by binding to E-box elements within the prompter region of their target genes (Huang et al., 2013). Expression of *Glyma.16g151500*, which encodes a NAC transcription factor, was significantly up-regulated during drought. The TF is a nuclear localized TF that regulates many important processes *via* gene regulation.

*Glyma.16g151500* was up-regulated 25 times in overexpression lines, compared with expression in the wild type, which was the greatest difference in expression among the predicted interaction genes (Figure 7B). The gene encodes a protein that contains a NAC domain and has DNA binding characteristics. The ERF proteins regulate biotic and abiotic stress responses by directly or indirectly regulating gene expression by binding with the GCC-box motif or by interacting with other TFs (Fujimoto et al., 2000; Zhu et al., 2003; Min et al., 2014). The tomato ERF protein TSRF1 binds to the *cis*-acting element GCC-box in the promoter of a pathogenesis-related gene and positively regulates pathogen resistance in tomato and tobacco (Fujimoto et al., 2000). The gene also increases expression of MYB, MYC, and proline synthesis-related genes, which contain the GCC-box in their promoter region, leading to improved osmotic and drought tolerance (Quan et al., 2010). Therefore, *cis*-elements and their associated genes may be important in regulating the response of soybean to drought stress (Liu et al., 1998).

## Conclusion

In this study, 301 *GmAP2/ERF* genes encoding TFs were identified in the soybean genome, which were unevenly distributed on 20 chromosomes and two chromosome scaffolds. The TFs were divided into five categories according to their homology. Then, according to results of previous studies, the target gene *GmAP2/ERF144* was selected from the genes up-regulated by drought and salt stress in the transcriptome. According to tissue expression analysis and subcellular determination, the gene was highly expressed in leaves, and its protein was localized in the nucleus. To validate gene function, *GmAP2/ERF144* was overexpressed in soybean using an *Agrobacterium*-mediated method. Compared with wild-type soybean, drought resistance in overexpression lines increased significantly. Network analysis was used to predict genes that interacted with *GmAP2/ERF144*. To conclude, this work provides a foundation to increase understanding of mechanisms of drought resistance in soybean and ultimately cultivation of drought-resistant varieties.

## Data Availability Statement

The original contributions presented in the study are included in the article/Supplementary Material, further inquiries can be directed to the corresponding author/s.

## Author Contributions

HW, JZ, and HXi designed the experiments and wrote the manuscript. HW, DN, JS, SD, CW, and HXu executed the experiments and prepared the figures. HW, JX, LZ, and NG analyzed the data. All authors read and approved the final manuscript.

## Conflict of Interest

The authors declare that the research was conducted in the absence of any commercial or financial relationships that could be construed as a potential conflict of interest.

## Publisher’s Note

All claims expressed in this article are solely those of the authors and do not necessarily represent those of their affiliated organizations, or those of the publisher, the editors and the reviewers. Any product that may be evaluated in this article, or claim that may be made by its manufacturer, is not guaranteed or endorsed by the publisher.

## Footnotes

1
https://smart.embl-heidelberf.de/

2
http://expasy.org/tools/

3
http://cello.life.nctu.edu.tw/

4
http://meme-suite.org/tools/meme

5
https://mafft.cbrc.jp/alignment/server/

6
http://nls-mapper.iab.keio.ac.jp/cgi-bin/NLS_Mapper_form.cgi

7
https://string_vector_graphic.SVG

8
https://soybase.org/

9
http://nls-mapper.iab.keio.ac.jp/cgi-bin/NLS_Mapper_form.cgi

## Supplementary Material

Supplementary Figure 1 Phylogenetic relations, gene structures, and motif patterns of *GmAP2/ERF* members. Phylogenetic relations of the **(A)** *AP2* subfamily, **(B)** *ERF* subfamily, **(C)** *DREB* subfamily, and **(D)** *RAV* subfamily. Orange boxes, blue boxes, and black lines indicate UTRs, exons, and introns, respectively. Bottom scales indicate exon and intron lengths.

Supplementary Figure 2 Cis-Element Analyses of Soybean *GmAP2/ERF* Genes. Therefore, the promoter region sequences (the 2-kp upstream sequences from gene initiation codons) of *GmAP2/ERF* genes were extracted for *cis*-element analyses. *Cis*-elements in the 2,000-bp upstream regions of soybean *GmAP2/ERF* genes were predicted using the PlantCare database, and colored boxes contain their names and positions (relative to the start codon).

Supplementary Figure 3 Salt-responsive differentially expressed genes and expression profiles of soybean *GmAP2/ERF* genes. Heat map with hierarchical cluster analysis of salt-responsive differentially expressed genes (DEGs) among soybean *GmAP2/ERF* genes. Different colors indicate relative transcript abundance of salt-responsive soybean *GmAP2/ERF* genes, with red indicating high abundance and green indicating low abundance.

Supplementary Figure 4 GmAP2/ERF144 protein structure analysis.

Supplementary Figure 5 Identification of *GmAP2/ERF144* transgenic lines. **(A)** Cloning of *GmAP2/ERF144*. **(B)** Quantitative real-time (RT)-PCR analysis of four independent transgenic soybean plants overexpressing *GmAP2/ERF144*. **(C)** Herbicide screening transgenic lines. **(D)** The transgenic positive vaccine was detected by PCR. **(E)** Detection of overexpression soybean herbicide bar marker. Data represent the mean ± SE. *, *P* < 0.05 (Student’s *t*-test). ^**^, *P* < 0.01 (Student’s *t*-test).
