# Supplementary figures and images for: Genome-Wide Identification of the AP2/ERF Gene Family and Functional Analysis of GmAP2/ERF144 for Drought Tolerance in Soybean
Source: Front Plant Sci. 2022 Mar 28;13:848766. doi: 10.3389/fpls.2022.848766 (PMC8996232; doi:10.3389/fpls.2022.848766)

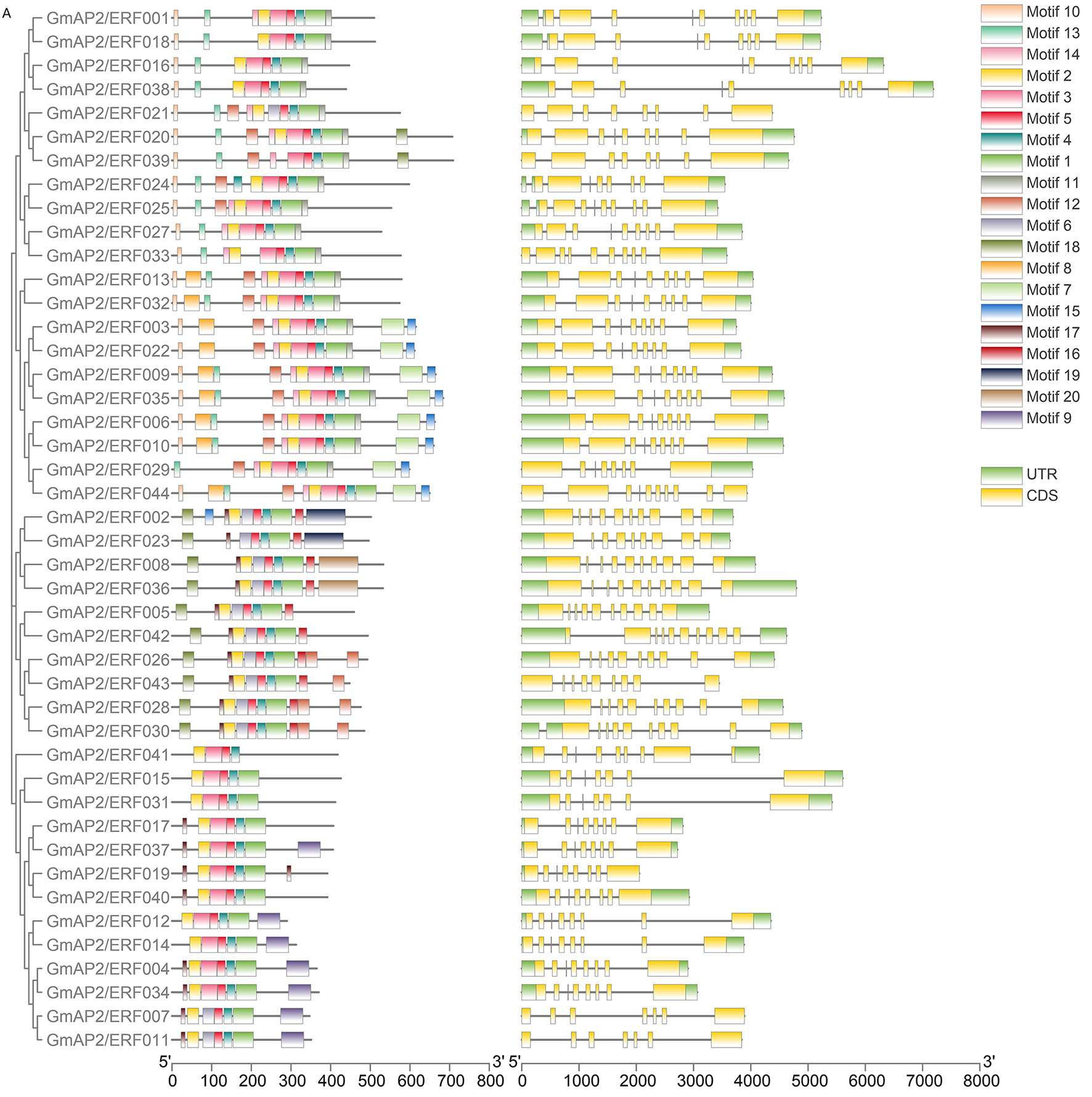

Supplement: Supplementary Figure 1 — Phylogenetic relations, gene structures, and motif patterns of GmAP2/ERF members. Phylogenetic relations of the (A) AP2 subfamily, (B) ERF subfamily, (C) DREB subfamily, and (D) RAV subfamily. Orange boxes, blue boxes, and black lines indicate UTRs, exons, and introns, respectively. Bottom scales indicate exon and intron lengths. [file Image_1.TIF]

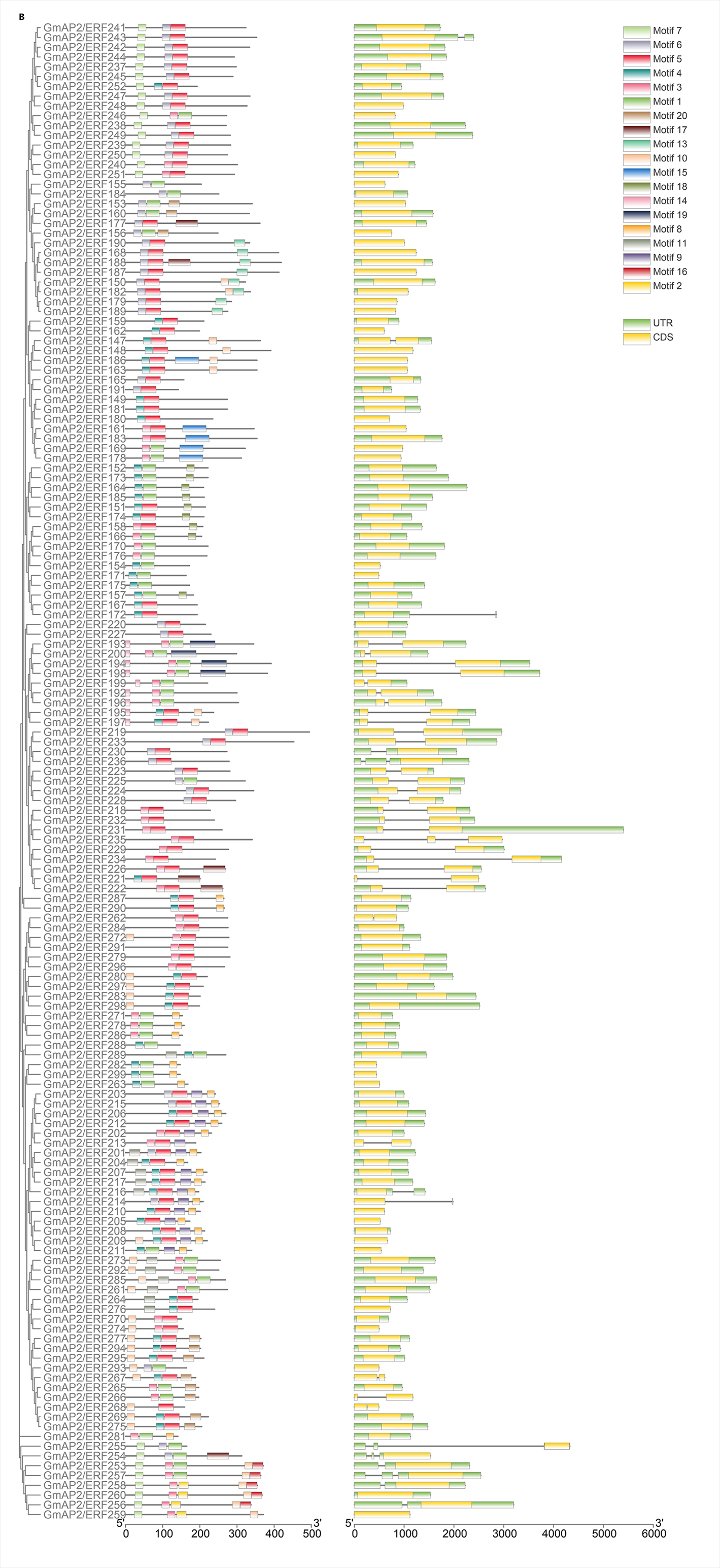

Supplement: Supplementary Figure 2 — Cis-Element Analyses of Soybean GmAP2/ERF Genes. Therefore, the promoter region sequences (the 2-kp upstream sequences from gene initiation codons) of GmAP2/ERF genes were extracted for cis-element analyses. Cis-elements in the 2,000-bp upstream regions of soybean GmAP2/ERF genes were predicted using the PlantCare database, and colored boxes contain their names and positions (relative to the start codon). [file Image_2.TIF]

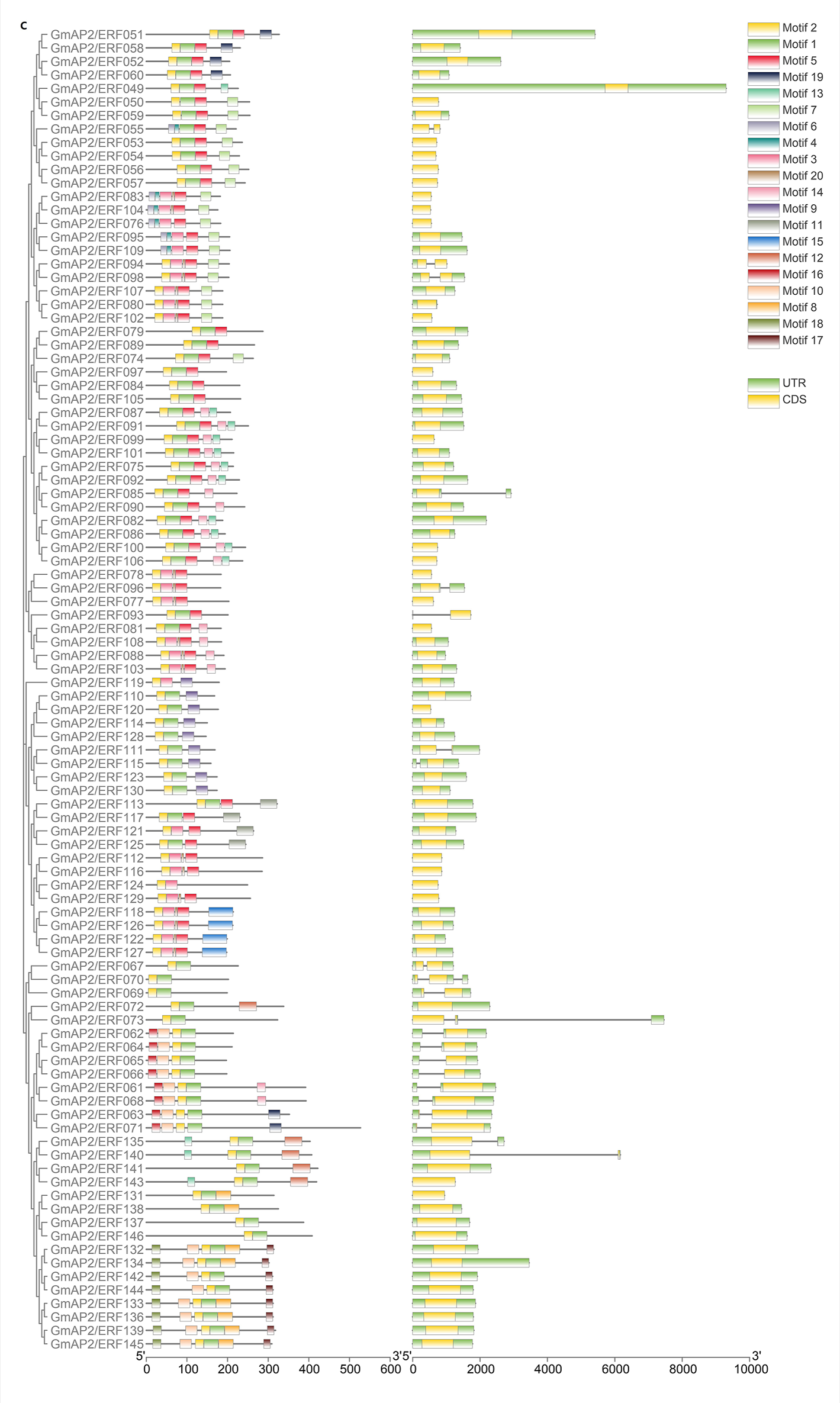

Supplement: Supplementary Figure 3 — Salt-responsive differentially expressed genes and expression profiles of soybean GmAP2/ERF genes. Heat map with hierarchical cluster analysis of salt-responsive differentially expressed genes (DEGs) among soybean GmAP2/ERF genes. Different colors indicate relative transcript abundance of salt-responsive soybean GmAP2/ERF genes, with red indicating high abundance and green indicating low abundance. [file Image_3.TIF]

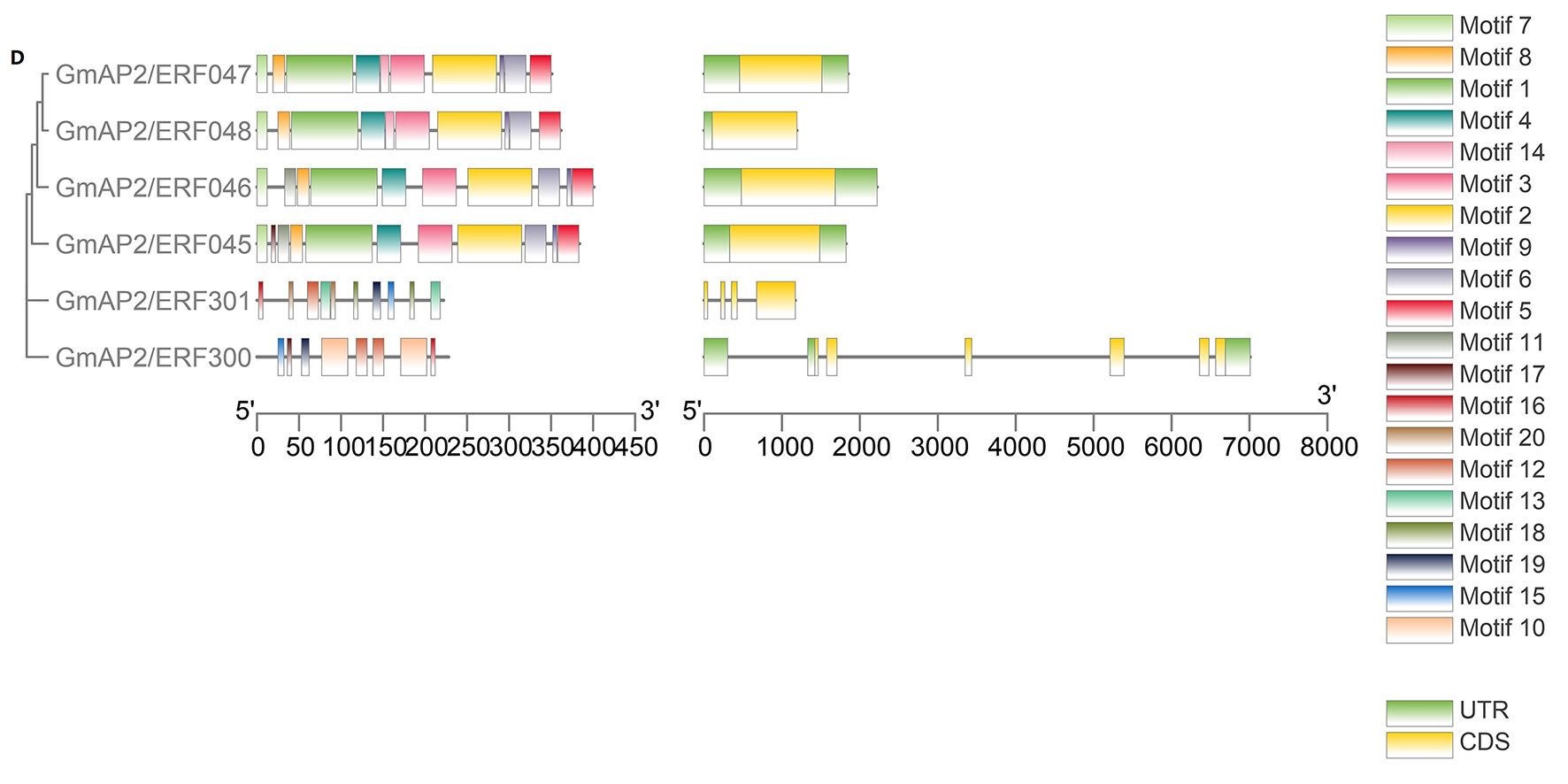

Supplement: Supplementary Figure 4 — GmAP2/ERF144 protein structure analysis. [file Image_4.TIF]

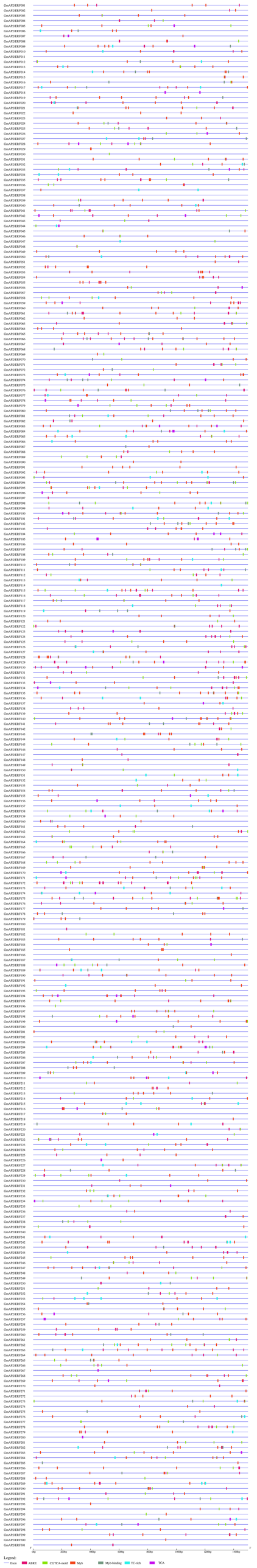

Supplement: Supplementary Figure 5 — Identification of GmAP2/ERF144 transgenic lines. (A) Cloning of GmAP2/ERF144. (B) Quantitative real-time (RT)-PCR analysis of four independent transgenic soybean plants overexpressing GmAP2/ERF144. (C) Herbicide screening transgenic lines. (D) The transgenic positive vaccine was detected by PCR. (E) Detection of overexpression soybean herbicide bar marker. Data represent the mean ± SE. *, P < 0.05 (Student’s t-test). **, P < 0.01 (Student’s t-test). [file Image_5.TIF]

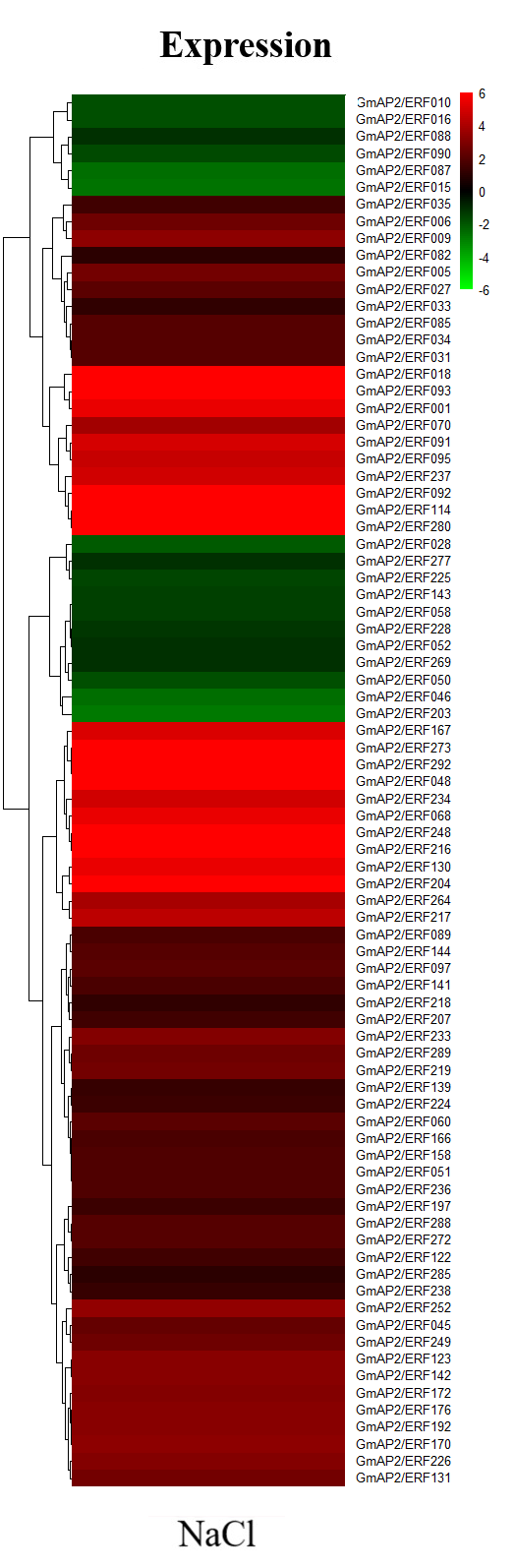

Supplement: Supplementary file 6 [file Image_6.TIF]

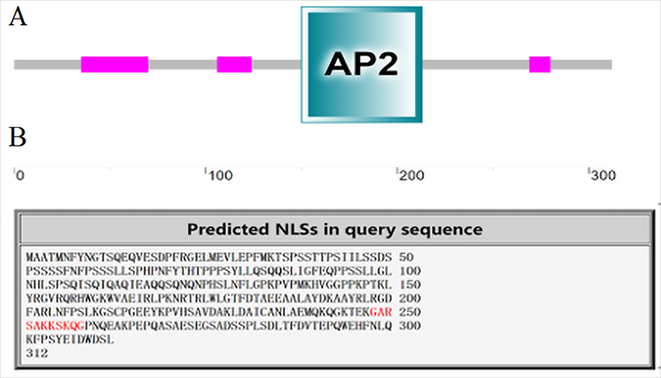

Supplement: Supplementary file 7 [file Image_7.TIF]

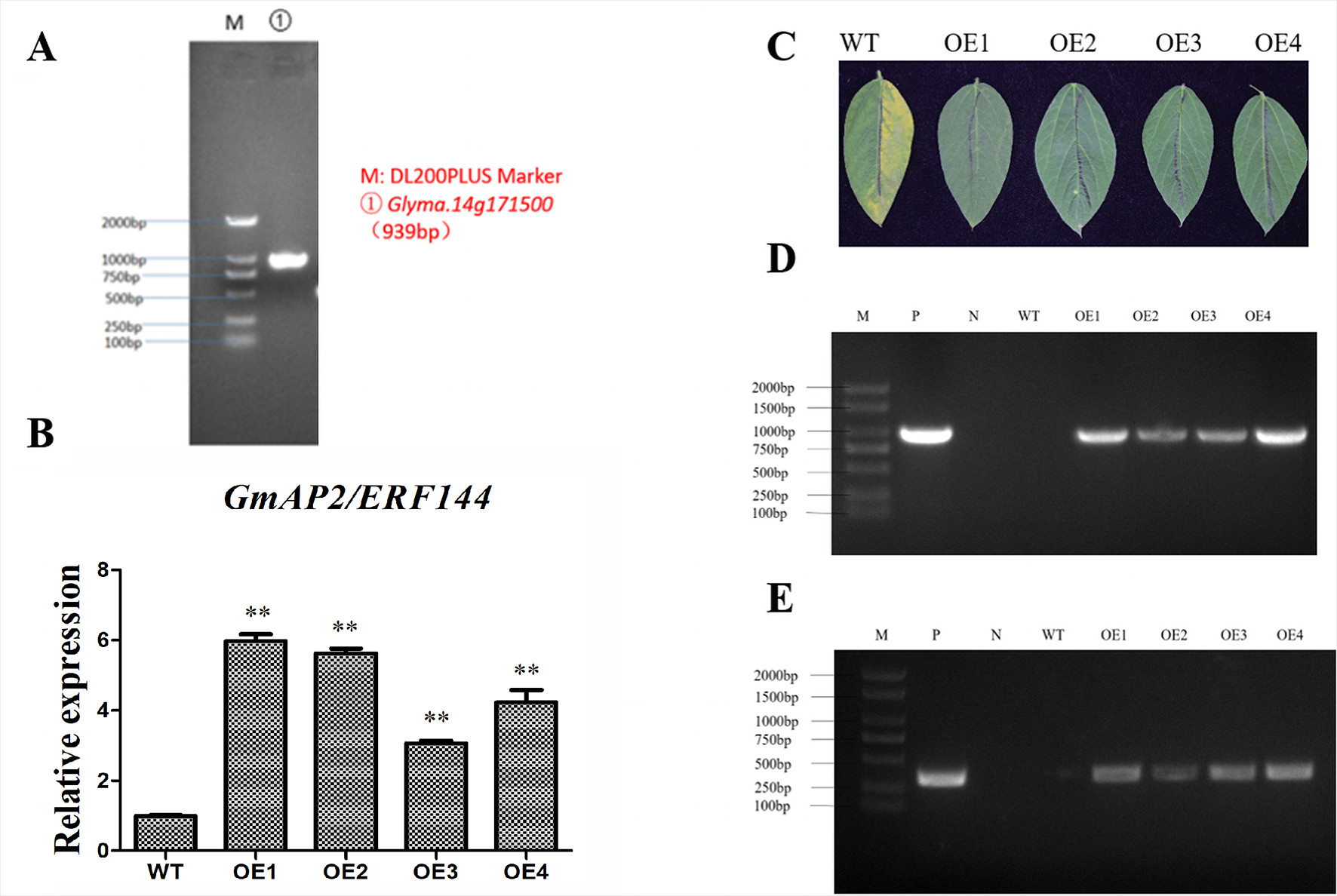

Supplement: Supplementary file 8 [file Image_8.TIF]
